# Analogues of Disulfides from *Allium stipitatum* Demonstrate Potent Anti-tubercular Activities through Drug Efflux Pump and Biofilm Inhibition

**DOI:** 10.1038/s41598-017-18948-w

**Published:** 2018-01-18

**Authors:** Cynthia A. Danquah, Eleftheria Kakagianni, Proma Khondkar, Arundhati Maitra, Mukhlesur Rahman, Dimitrios Evangelopoulos, Timothy D. McHugh, Paul Stapleton, John Malkinson, Sanjib Bhakta, Simon Gibbons

**Affiliations:** 1 Research Department of Pharmaceutical and Biological Chemistry, UCL School of Pharmacy, 29-39 Brunswick Square, London, WC1N 1AX UK; 2 Department of Biological Sciences, Institute of Structural and Molecular Biology, Birkbeck, University of London, Malet Street, London, WC1E 7HX UK; 3 Department of Pharmaceutical, Chemical and Environmental Sciences, University of Greenwich, Central Avenue, Chatham Maritime, ME4 4TB UK; 4 Medicine Research Group, School of Health, Sport and Bioscience, University of East London, Water Lane, London, E15 4LZ UK; 5 Centre for Clinical Microbiology, UCL Royal Free Hospital, Rowland Hill, London, NW3 2PF UK

## Abstract

Disulfides from *Allium stipitatum*, commonly known as Persian shallot, were previously reported to possess antibacterial properties. Analogues of these compounds, produced by *S*-methylthiolation of appropriate thiols using *S*-methyl methanethiosulfonate, exhibited antimicrobial activity, with one compound inhibiting the growth of *Mycobacterium tuberculosis* at 17 µM (4 mg L^−1^) and other compounds inhibiting *Escherichia coli* and multi-drug-resistant (MDR) *Staphylococcus aureus* at concentrations ranging between 32–138 µM (8–32 mg L^−1^). These compounds also displayed moderate inhibitory effects on *Klebsiella* and *Proteus* species. Whole-cell phenotypic bioassays such as the spot-culture growth inhibition assay (SPOTi), drug efflux inhibition, biofilm inhibition and cytotoxicity assays were used to evaluate these compounds. Of particular note was their ability to inhibit mycobacterial drug efflux and biofilm formation, while maintaining a high selectivity towards *M*. *tuberculosis* H37Rv. These results suggest that methyl disulfides are novel scaffolds which could lead to the development of new drugs against tuberculosis (TB).

## Introduction

**Figure 1:**
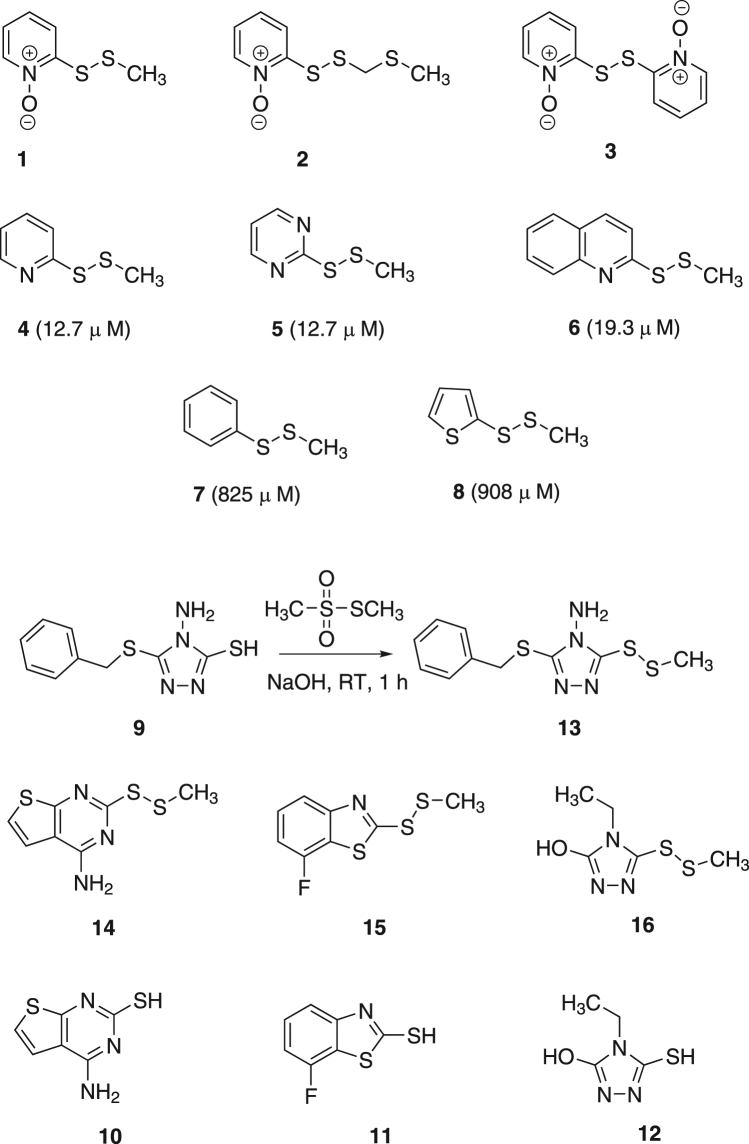
Compounds isolated from *Allium stipitatum* with antibacterial activity (**1**–**3**). Synthesized compounds (**4**–**8**) based on the natural products. MIC values against *S*. *aureus* are in parentheses. Reaction scheme for the synthesis of compounds (**13–16**) and the resulting synthesized methyl disulfides.

We investigated extracts of bulbs from the plant family Alliaceae for their ability to produce antibacterial compounds, and from *Allium neapolitanum*, antibacterial canthinone alkaloids and hydroxy acids were characterised^1^. Of more chemical and pharmacological interest, a study on the Central Asian species *Allium stipitatum*, led to the isolation of three novel pyridine-*N*-oxide alkaloids (**1**–**3**), displaying outstanding potency towards *Mycobacterium tuberculosis* (Fig. 1)^2^. The minimum inhibitory concentrations (MIC) exhibited by these compounds were clinically-relevant and found to range between 2.5–40 µM (0.5–8 mg L^−1^). Subsequently, a series of structurally-related methyl disulfides were synthesized in an effort to optimize the exceptional antibacterial activity. Structure-activity relationships revealed that the presence of the disulfide moiety was not the only factor responsible for activity, and it is possible that the disulfide is strongly “activated” by the presence of electron-withdrawing functional groups such as pyridine, pyridine-*N*-oxide, pyrimidine and quinoline, whereas phenyl and thiophene were poorly electron withdrawing and therefore had little effect on the “reactivity” of the disulfide bond (Fig. 1)^2^. From compounds **4–6**, it was clear that the *N*-oxide was not a prerequisite for antibacterial activity. Based on this rationale, we synthesised a small set of disulphides with proximal electron-withdrawing groups and characterised their antibacterial properties.

Given the continuing issues of multidrug-resistant (MDR) and extensively-drug-resistant (XDR) cases that are increasingly associated with clinically-relevant Gram-positive, Gram-negative and acid-fast human pathogens (such as *Staphylococcus aureus*, *Escherichia coli* and *Mycobacterium tuberculosis* respectively), there is a pressing need to develop new classes of antibacterials^3–5^. Common strategies for effective antimicrobial development are to target novel endogenous effector machinery within a pathogen or to reverse resistance and thereby make the bacteria more susceptible to existing chemotherapy. Increased levels of tolerance towards drugs are observed in bacteria that contain systems to prevent these compounds from reaching their site(s) of action^6^. Within this paradigm, efflux pump-related multidrug-resistance significantly contributes to a reduction in drug accumulation and often renders antibiotics redundant^7^. This could be circumvented by molecules that interfere with or inhibit antibiotic efflux^8,9^. Additionally, multidrug efflux pumps are often transmembrane proteins that secrete metabolites involved in quorum-sensing^10^. This *cross-talk* between bacteria is believed to be essential for the formation and dispersion of bacterial biofilms^11^. Therefore, inhibition of multidrug efflux pumps is also a strategy to inhibit biofilm formation, which is a major contributor to antimicrobial resistance^11^.

The aim of this study was to synthesise the novel disulphide compounds mentioned earlier and comprehensively evaluate their biological activity to optimise the chemical scaffold as a prospective therapeutic lead.

## Results

### Synthesis of the antibacterial methyl disulfides

To probe the antibacterial potency, efflux and biofilm inhibitory properties, we chose an initial series of aromatic and heterocyclic thiols on the basis of their commercial availability, namely 4-amino-5-(benzylthio)-4*H*-1,2,4-triazole-3-thiol (**9**), 4-aminothieno[2,3-*d*]pyrimidine-2-thiol (**10**), 7-fluorobenzo[*d*]thiazole-2-thiol (**11**) and 4-ethyl-5-mercapto-4*H*-1,2,4-triazol-3-ol (**12**). Each aromatic thiol was treated with *S*-methyl methanethiosulfonate under alkaline conditions to generate the methyl disulfides, compounds **13**–**16** (Fig. 1 and Supplementary Information).

### Antibacterial Bioassay of the methyl disulfides

The spot culture growth inhibition (SPOTi) assay is a whole-cell phenotypic screen that is routinely used to identify novel antimicrobial molecules with clinical relevance^12,13^. This rapid but gold-standard assay was applied to evaluate the antimicrobial activity of the synthesized compounds against Gram-positive, Gram-negative and acid-fast bacteria. All of the synthesized methyl disulfides demonstrated antibacterial activity to varying extents (Table 1). Based on the encouraging results when tested against the non-pathogenic model of *M*. *tuberculosis* organisms, *M*. *aurum* (ATCC23366) and *M*. *bovis* BCG (ATCC35734), the compounds were subsequently tested against *M*. *tuberculosis* H37Rv and its multidrug-resistant clinical isolates (Mtb-MDR1 and Mtb-MDR2). All four compounds showed anti-mycobacterial activities when tested, with compound **14** having the lowest MIC of 17 µM (4 mg L^−1^), against the virulent *M*. *tuberculosis* H37R_V_. Additionally, compounds **13**–**16** exhibited antibacterial activity against the Gram-positive *Staphylococcus aureus* strains (including effluxing multidrug-resistant strains) and *Enterococcus faecalis*. In particular, compounds **14** and **16** were active against *S*. *aureus* with MIC values ranging between 70–84 µM (16 mg L^−1^).

**Table 1:** Minimum Inhibitory Concentrations (MIC) in µM (mg L^−1^) of the synthesized compounds (**13**–**16**) against non-pathogenic mycobacteria and pathogenic multidrug-resistant clinical isolates of *Mycobacterium tuberculosis*, as well as Gram-positive and Gram-negative bacteria.

**Compound**	*M*. *smegmatis*	*M*. *aurum*	*M*. *bovis BCG*	*M*. *tuberculosis H37Rv*	*M*. *tuberculosis MDR1*	*M*. *tuberculosis MDR2*	*E*. *coli (NCTC 10418)*	*Proteus mirabilis -10830*	*K*. *pneumoniae*	*S*. *aureus SA-1199B*	*S*. *aureus XU212*	**EMRSA-15**	*Ent*. *faecalis*
**13**	113 (32)	113 (32)	113 (32)	225 (64)	450 (128)	898 (256)	450 (128)	450 (128)	450 (128)	113 (32)	56 (16)	225 (64)	113 (32)
**14**	70 (16)	70 (16)	70 (16)	17 (4)	70 (16)	140 (32)	558 (128)	2232 (512)	2232 (512)	70 (16)	70 (16)	70 (16)	140 (32)
**15**	277 (64)	277 (64)	277 (64)	138 (32)	>2213 (>512)	>2213 (>512)	553 (128)	2213 (512)	>2213 (>512)	138 (32)	69 (16)	69 (16)	69 (16)
**16**	84 (16)	84 (16)	84 (16)	167 (32)	167 (32)	669 (128)	84 (16)	335 (64)	335 (64)	84 (16)	84 (16)	42 (8)	84 (16)
**Norfloxacin**	—	—	—	—	—	—	0.4 (0.125)	>200 (>64)	>200 (>64)	200 (64)	25 (8)	2 (0.5)	6 (2)
**Isoniazid**	29 (4)	4 (0.5)	0.7 (0.1)	0.7 (0.1)	0.7 (0.1)	0.7 (0.1)	—	—	—	—	—	—	—
**Rifampicin**	10 (8)	0.1 (0.1)	0.6 (0.5)	0.1 (0.1)	—	—	—	—	—	—	—	—	—

### Efflux Pump Inhibitory Activity

**Figure 2:**
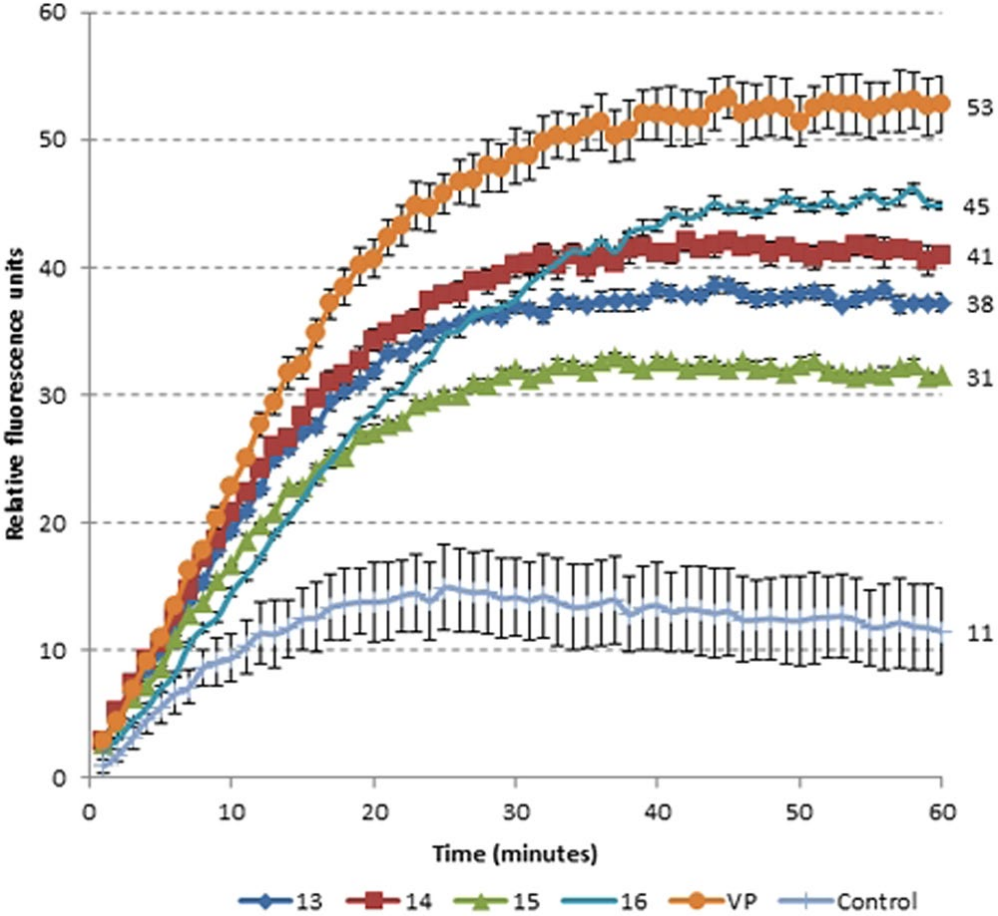
Efflux pump inhibition (EPI) of *M*. *aurum* under the pressure of methyl disulfides **13**–**16**. Ethidium bromide (EtBr), an efflux pump substrate was used at a final concentration of 1.3 µM (0.5 mg L^−1^). Its accumulation within the bacterial cells is an indicator of disruption of the efflux mechanism and was detected using fluorescence emissions. Verapamil (VP), a known efflux pump inhibitor, and a drug-free culture were used as positive and negative controls respectively. Low (11–20 rfu) to very high (>50 rfu) inhibition of efflux are represented by the numbers at the side of the graph. The experiments were performed in triplicate (*n* = 3) and the graph was plotted using the averages. (rfu = relative fluorescence units).

Multi-drug efflux pumps are a key mechanism through which many pathogens, *M*. *tuberculosis* in particular, develop intrinsic resistance or tolerance towards xenobiotic compounds^14,15^. Ethidium bromide (EtBr) is a known substrate for these pumps and its accumulation inside the bacterial cell, when the extrusion mechanism is impaired, can be followed by detecting its fluorescence^16^. EtBr is usually quenched in an aqueous environment and fluoresces when interacting with the hydrophobic regions within the bacilli^17^. Verapamil, a calcium channel blocker, is widely used as an inhibitor of efflux in mycobacterial cells and was used as a control in our experiments^15^. All of the compounds showed inhibition of efflux in the whole-cell model (Fig. 2), with compound **14** and **16** being the most active inhibitors, without affecting the cell viability (a concentration of 25% of the MIC was used for the assay).

### Methyl disulfides as bacterial biofilm inhibitors

As alluded to earlier, efflux mechanisms are involved in quorum-sensing that in turn plays a pivotal role in biofilm formation^11^. The transcriptional activator LuxR is heavily implicated in quorum sensing and induction of biofilm formation in a variety of bacteria, and is also found in *M*. *tuberculosis* and *M*. *leprae*^18,19^. Tubercle bacilli have a natural tendency to form biofilms and other multi-cellular structures, known as cords in liquid culture^20^. Multi-cellular aggregates resembling biofilms have been detected in the acellular rims of granulomas and necrotic lesions^21^. Other species belonging to the *M*. *tuberculosis* complex (MTBC) such as *M*. *avium* are known to form stable biofilms in water reservoirs and can invade lung tissues^22^. The ability to form cords and biofilms has been correlated with the pathogen’s virulence^22^. Biofilm-deficient mutants of the pathogen show reduced ability to invade epithelial cells as well as to cause infection in mouse models^19^.

**Figure 3:**
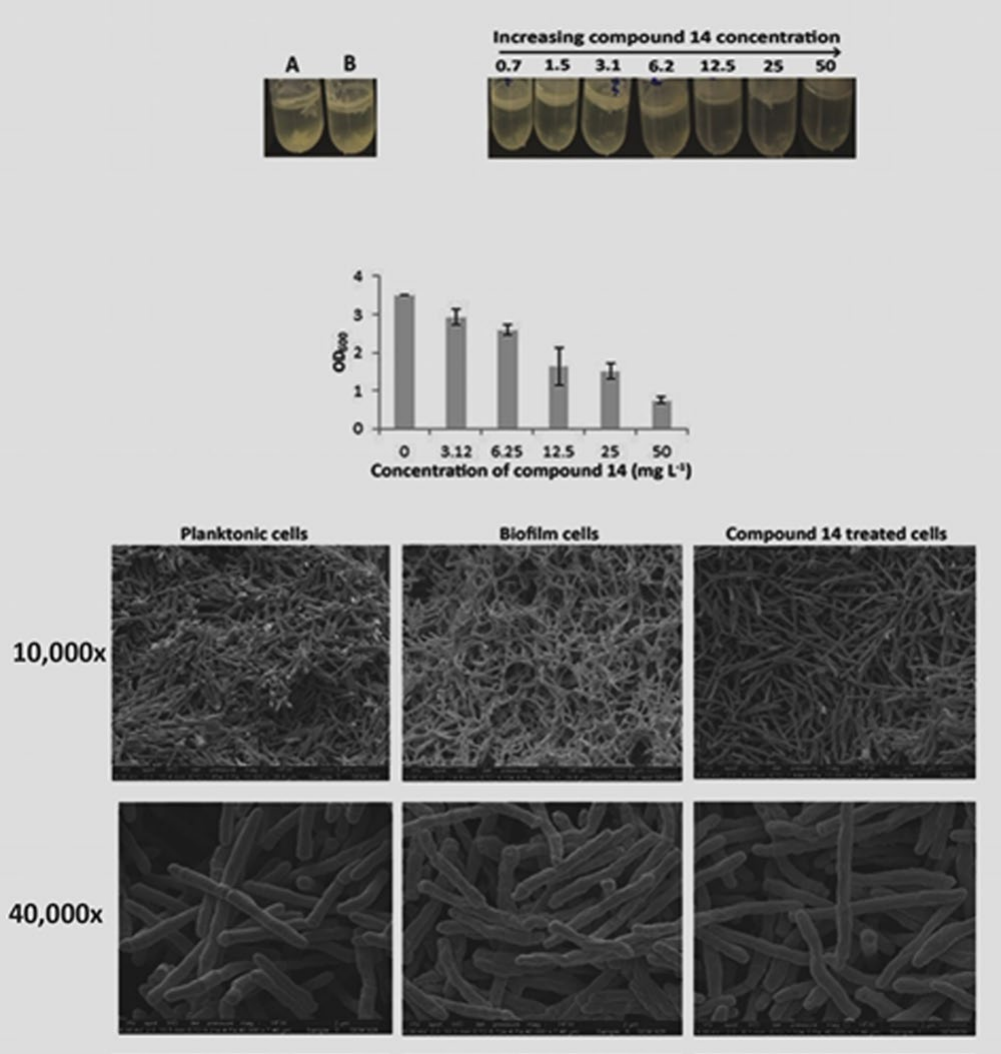
Inhibition of *M*. *smegmatis* biofilm formation in the presence of varying concentrations of compound **14**. (**a**) Dose-dependent inhibition of *M*. *smegmatis* biofilm formation, as observed by their thinning in the presence of compound **14**. Tubes A and B are ‘no drug’ and solvent (0.1% DMSO) controls respectively. Note that the biofilm formation initiated at the air-liquid interface in *M*. *smegmatis*. A newly-formed biofilm becomes stacked on top of the old layer and generates a downwards push. Once a critical biomass was exceeded, the lower part of the mature biofilm was observed to dissociate and settle at the bottom of the stand-culture-tube (see controls in which no inhibitor was added). (**b**) Crystal violet staining of the biofilms showing a decrease in the intensity of the stain with increasing concentrations of compound **14**. (**c**) SEM images of *M*. *smegmatis* planktonic, untreated biofilms and biofilms treated with 50 mg L^−1^ of compound **14**.

*M*. *smegmatis*, a non-pathogenic model for *M*. *tuberculosis*, forms stable biofilms at the liquid-air interface within 5 days and was used to test whether the impairment of drug efflux could also inhibit the formation of biofilms in mycobacteria^15^. As compound **14** was found to be the most potent anti-mycobacterial (see Table 1), it was selected for the biofilm inhibition studies. Compound **14** was observed to inhibit the growth of *M*. *smegmatis* biofilms in a concentration-dependent manner even at sub-MIC levels (Fig. 3a and b) when compared to controls. This finding was further validated through a quantitative crystal violet staining method^23^. Scanning electron microscopic^24^ images (Fig. 3c) of *M*. *smegmatis* biofilms revealed a dense lattice-like network of bacterial cells with rough outer coats that are likely to be composed of extracellular polymeric substances (EPS) such as lipids, proteins and extra-cellular DNA. On treatment with compound **14**, the outer layer of the bacilli became smoother and they appeared to lose the mesh-like inter-cellular connections within the community.

### Selectivity

The synthesized compounds showed a range of eukaryotic toxicity profiles against murine macrophage RAW264.7 cells (Table 2). Compound **14** demonstrated a promising SI of 16.

**Table 2:** Cytotoxicity profile of compounds **13**–**16** and selectivity against the murine macrophage cell line RAW 264.7 using the resazurin assay.

Compound	MIC^[a]^*M*. *tb* H37Rv µM (mg L^−1^)	GIC ^[b]^µM (mg L^−1^)	SI^[c]^
**13**	225 (64)	439 (125)	1.95
**14**	17 (4)	272 (62.5)	16
**15**	138 (32)	135 (31.3)	0.98
**16**	167 (32)	40 (7.8)	0.24
**INH**	0.7 (0.1)	No inhibition	*

INH was used as a control drug and shows no effect on the viability of the cells. A drop in the fluorescence levels indicate loss of viability of cells as determined by the reduction in the oxidation of resazurin to resorufin which in turn fluoresces. The experiments were performed in triplicate. ^[a]^MIC - minimum inhibitory concentration.

^[b]^GIC - growth inhibitory concentration.

^[c]^SI - selectivity index, where SI = GIC/MIC (SI calculated using the µM values in Table 2).

^[d]^INH- Isoniazid (control, front-line anti-tubercular drug)*. As no significant inhibition is observed the SI in these cases cannot be calculated.

## Discussion

The multidrug-resistant *S*. *aureus* SA-1199B (a strain that overexpresses NorA, a multidrug efflux transporter), proved to be as susceptible to the methyl disulfides as other non-NorA *S*. *aureus* isolates (Table 1). This indicated that the methyl disulfides may have a mechanism of action that evades NorA-mediated multi-drug efflux.

Interestingly, compound **16** inhibited the Gram-negative bacteria *Klebsiella pneumoniae* and *Proteus mirabilis* at an MIC of 335 µM (64 mg L^−1^) and whilst this is a moderate activity, it is rare to find compounds demonstrating antibacterial activity toward these organisms. Even the standard antibiotic control used for the assay, norfloxacin, could only inhibit the growth of these organisms at a minimum inhibitory concentration higher than 200 µM (64 mg L^−1^). The synthesized methyl disulfides exhibited appreciable antibacterial activity against *E*. *coli*; particularly compound **16** had good antibacterial activity with an MIC value of 84 µM (16 mg L^−1^).

Overall, the methyl disulfides exhibited inhibitory effects against Gram-positive bacterial strains and acid-fast *Mycobacterium* species. However, their moderate activity against the selected Gram-negative bacteria provided further incentive to investigate the endogenous mechanism(s) of action of these compounds.

Compounds **14** and **16** exhibited whole-cell drug efflux pump inhibitory activities higher than **13** and **15**. Cells treated with the known efflux pump inhibitor verapamil and inhibitor-free cells were used as positive and negative controls in this assay respectively (see Fig. 2).

In terms of the effects of the compounds on *Mycobacterium smegmatis* biofilm formation, the ability of compound **14** to concentration-dependently inhibit biofilm formation, even at sub-MIC levels is particularly noteworthy.

The efflux pump and biofilm inhibitory effects indicate the possible mechanisms of action of these compounds. This route of antibacterial activity against *Pseudomonas aeruginosa* was also noted by Jakobsen *et al*. (2012) for similar compounds^25^. In addition, allicin, one of the major volatile compounds present in garlic and also a disulfide has been reported to act through permeabilization of cell membranes and inactivation of metabolic enzymes resulting in depletion of intracellular glutathione pools^26,27^. Our ongoing genomic and transcriptomic analyses of bacterial cells under pressure of inhibitor compounds, as well as spontaneous resistant mutants followed by molecular and biochemical investigations of the relevant genes and their recombinant products should provide a deeper insight into the molecular mechanism(s) of action of these compounds.

For compounds **13**, **15** and **16**, the bacterial growth inhibition and macrophage cytotoxicity were similar, indicating poor selectivity for their antibacterial action (Table 2). However for compound **14**, the SI was 16. The SI provides information on the therapeutic potential of compounds as a function of the concentration range at which they are active against pathogenic mycobacteria while remaining non-toxic to mammalian cells. This provides information on the therapeutic potential of these compound, as a function of the concentration range at which it is active against the growth of pathogenic mycobacteria while remaining non-toxic to mammalian cells (murine macrophages in this case). In conclusion, these synthesized methyl disulfides are new chemical scaffolds that have potential as templates for the discovery of new anti-tubercular leads.

## Methods

### Materials and synthesis of methyl disulfides

Aromatic thiols 4-amino-5-(benzylthio)-4*H*-1,2,4-triazole-3-thiol (**9**), 4-aminothieno[2,3-*d*]pyrimidine-2-thiol (**10**), 7-fluorobenzo[*d*]thiazole-2-thiol (**11**), 4-ethyl-5-mercapto-4*H*-1,2,4-triazol-3-ol (**12**) were purchased from Sigma-Aldrich, Gillingham, U.K. The method of Kitson and Loomes (1985), for the synthesis of methyl 2- and 4-pyridyl disulfide from 2- and 4-thiopyridone and methyl methanethiosulfonate was adapted and modified as follows. The appropriate thiol (2.5 mmol) was dissolved in water (5 mL) containing NaOH (0.10 g, 2.5 mmol, 1 equiv.) and *S*-methyl methanethiosulfonate (0.315 g, 2.5 mmol, 1 equiv.) added. The solution was stirred for 1 h at room temperature. The cloudy suspension formed was extracted with CH_2_Cl_2_ (20 mL). The organic phase was then dried with anhydrous sodium sulfate, filtered, and concentrated under reduced pressure to afford the pure disulfide which was subsequently characterized by spectroscopic techniques – NMR, MS, HRMS, UV and IR (Supplementary Information).

### Antibacterial assays (whole-cell phenotypic assays)

Minimum inhibitory concentrations (MIC) of the compounds against *Mycobacterium* strains were determined using the spot-culture growth inhibition assay (SPOTi)^12,13,28,29^. The lowest concentration at which mycobacterial growth was completely inhibited by the compound was observed directly. Isoniazid and rifampicin were used as antibiotic controls and the experiments repeated in triplicate.

The antibacterial activity of the compounds was tested against Gram-negative bacteria: *Klebsiella pneumoniae*, *Proteus mirabilis* (10830), *Escherichia coli* (NCTC 10418) and Gram-positive bacteria: *Enterococcus faecalis* (12697), methicillin-resistant *Staphylococcus aureus* strains (XU-212 and EMRSA-15) and multidrug-resistant *Staphylococcus aureus* strain SA-1199B using the microtitre broth dilution assay. Norfloxacin served as a positive control. The assay was performed in 96-well plates and each methyl disulfide was tested in quadruplicate to confirm the reliability and reproducibility of the data. The MIC was determined after the addition of 3-(4,5-dimethylthiazol-2-yl)-2,5-diphenyltetrazolium bromide (MTT) to the 96-well plates. Bacterial growth was indicated by a colour change from yellow to dark blue, which was visually observed. The MIC was recorded as the lowest concentration at which no growth was observed^13,30^.

### Cytotoxicity assay

Eukaryotic cell toxicity assay was carried out using RAW 264.7 macrophage cells, grown in complete RPMI-1640 medium supplemented with 2 mM l-glutamine and 10% heat-inactivated fetal bovine serum and 1% l-glutamine in a 25 cm^2^ vented, screw-cap cell-culture flask (Flowgen Bioscience Ltd., Hessle, UK) and incubated at 37 °C with a supply of 5% CO_2_ until confluent growth was observed. Cytotoxicity of the compounds towards the murine macrophages was determined using the resazurin assay^29^. For quantitative analysis, the fluorescence intensity was measured at *λ*_ex_560/*λ*_em_590 nm using a FLUOstar OPTIMA micro plate reader. The growth inhibitory concentration (GIC) was reported as the lowest concentration of compound at which no viable eukaryotic cells were detected.

### Efflux pump inhibition assay

Efflux pump inhibition assays were performed following previously published protocols and modified using *M*. *aurum* cells^9,15,31^. The effect of the synthesized compounds and verapamil (positive control) on the accumulation of ethidium bromide (EtBr) was determined by measuring fluorescence using a fluorimeter (FLUOstar OPTIMA, BMG Labtech) and fluorescence data was acquired every 60 s for a total period of 60 min. The compounds were used at one quarter of their MICs and EtBr (a known efflux pump substrate) at a concentration of 1.3 µM (0.5 mg L^−1^).

### Biofilm assay (inhibition of biofilm formation)

A late log-phase (OD_600_ = 3.0) culture of *Mycobacterium smegmatis* was inoculated into Sauton’s media as 1:100 dilutions. This preparation (2 mL) was transferred into polypropylene tubes and a range of concentrations of compound **14** 3–218 µM (0.7–50 mg L^−1^) was then added to each tube. The cap was tightly closed to avoid evaporation of media and the cultures were incubated at 37 °C in a stationary incubator for 5 days. Tubes containing the diluted cultures without any compounds served as inhibitor-free controls and those with only DMSO served as solvent controls. After 5 days, the biofilm samples were observed using the scanning electron microscope^24^. The experiments were performed in triplicate.

Once the biofilms were formed, the medium containing planktonic cells was removed carefully using a hypodermic needle. 1% crystal violet was added to the tubes so as to cover the biofilm and was left for 10 min. The crystal violet solution was discarded and the tubes were washed at least three times until no further stain was present in the washings. Ethanol (95% *v/v* in water) was then added to the tubes and left for 10 min. The solutions were then diluted 1:3 with ethanol and the absorbance of each was measured at 600 nm.

The SEM images were analysed with ImageJ (NIH) software^32^. Each image was calibrated individually and measurements were recorded for at least 200 cells for each condition from a minimum of five fields with varying magnifications.

## Electronic supplementary material

Supplementary Information
