# Supplementary material for: Analogues of Disulfides from Allium stipitatum Demonstrate Potent Anti-tubercular Activities through Drug Efflux Pump and Biofilm Inhibition
Source: Sci Rep. 2018 Jan 18;8:1150. doi: 10.1038/s41598-017-18948-w (PMC5773482; doi:10.1038/s41598-017-18948-w)
Supplement: Supplementary file 1 — Supplementary Information [file 41598_2017_18948_MOESM1_ESM.doc]

**Supplementary Information**

Analogues of Disulfides from *Allium stipitatum* Demonstrate Potent Anti-tubercular Activities through Drug Efflux Pump and Biofilm Inhibition

Cynthia A. Danquah^1,2^, Eleftheria Kakagianni^1^, Proma Khondkar^1,3^, Arundhati Maitra^2^, Mukhlesur Rahman^4^, Dimitrios Evangelopoulos^5^, Timothy D. McHugh^5^, Paul Stapleton^1^, John Malkinson^1^, Sanjib Bhakta^Δ2^, Simon Gibbons^Δ1^

^1^Research Department of Pharmaceutical and Biological Chemistry, UCL School of Pharmacy, 29-39 Brunswick Square, London WC1N 1AX, UK

^2^Department of Biological Sciences, ISMB, Birkbeck University of London, Malet Street, London WC1E 7HX, UK

^3^Department of Pharmaceutical, Chemical and Environmental Sciences, University of Greenwich, Central Avenue, Chatham Maritime, ME4 4TB, UK

^4^Sport and Bioscience Institution, University of East London, Water Lane, London E15 4LZ, UK

^5^Centre for Clinical Microbiology, UCL Royal Free Hospital, Rowland Hill, London NW3 2PF, UK

^Δ^These authors contributed equally to this work

Correspondence and requests for materials should be addressed to S. Gibbons (email: simon.gibbons@ucl.ac.uk) and S. Bhakta (email: [s.bhakta@bbk.ac.uk](mailto:s.bhakta@bbk.ac.uk))

**2. Synthesized methyl disulfides**

1. 3-(Benzylthio)-5-(methyldisufanyl)-4*H*-1,2,4-triazol-4-amine **13**
2. 2-(Methyldisulfanyl)thieno[2,3-*d*]pyrimidin-4-amine **14**
3. 7-Fluoro-2-(methyldisulfanyl)benzo[*d*]thiazole **15**
4. 4-Ethyl-5-(methyldisulfanyl)-2,4-dihydro-3*H*-1,2,4-triazol-3-one **16**

**Physical, UV, IR, HRMS and NMR spectral data of compounds 13-16**

**3-(Benzylthio)-5-(methyldisulfanyl)-4*H*-1,2,4-triazol-4-amine (13)**

White solid; Amount obtained 0.115 g (40 %). UV (CHCl_3_) *λ*_max_ (log *ε*) 208.0 (3.75), 255.0 (3.93) nm; IR (film) *v*_max_ 3247, 3142, 3025, 2973, 2918, 1413, 1382, 775, 697, 679 cm^-1^; ^1^H NMR and ^13^C NMR (Table 3); HRESIMS *m/z* 285.0316 [M+H]^+^ (calculated for C_10_H_12_N_4_S_3_ 285.0320).

**2-(Methyldisulfanyl)thieno[2,3-*d*]pyrimidin-4-amine (14)**

Pale orange powder; Amount obtained 0.103 g (45 %). UV (MeOH) *λ*_max_ (log *ε*) 228.0 (4.29), 281.0 (3.93); IR (film) *v*_max_ 3068, 2983, 2912, 1556, 1470, 1408, 1271, 1238, 1032, 783 cm^-1^; ^1^H NMR and ^13^C NMR (Table 4); HRESIMS *m/z* 229.9888 [M+H]^+^ (calculated for C_7_H_7_N_3_S_3_ 229.9880).

**7-Fluoro-2-(methyldisulfanyl)benzo[*d*]thiazole (15)**

White fluffy solid; Amount obtained 0.109 g (47 %). UV (MeOH) *λ*_max_ (log *ε*) 218.0 (4.42), 271.0 (3.86); IR (film) *v*_max_ 3070, 2982, 2911, 1556, 1470, 1460, 1406, 1271, 1238, 1033, 781 cm^-1^; ^1^H NMR and ^13^C NMR (Table 5); HRESIMS *m/z* 231.9736 [M+H]^+^ (calculated for C_8_H_6_ FNS_3_ 231.9725).

**4-Ethyl-5-(methyldisulfanyl)-4*H*-1,2,4-triazol-3-ol (16)**

Pale yellow powder; Amount obtained 0.079 g (41 %). UV (MeOH) *λ*_max_ (log *ε*) 210.0 (3.08); IR (film) *v*_max_ 3087, 3016, 2950, 2860, 2770, 2617, 2312, 1686, 1489, 1305, 774, 749, 683 cm^-1^; ^1^H NMR and ^13^C NMR (Table 7); HRESIMS *m/z* 192.0261 [M+H]^+^ (calculated for C_5_H_9_N_3_OS_2_ 192.0265).

Table 3. ^1^H NMR (500 MHz) and ^13^C NMR (125 MHz)

spectral data for compound **13** in (CD_3_)_2_SO

| **Position** | **^1^H** | **^13^C** | **HMBC** | |
| --- | --- | --- | --- | --- |
|  |  |  | ***^2^J*** | ***^3^J*** |
| 3 | - | 152.0 | - | - |
| 5 | - | 154.0 | - | - |
| 1’ | 4.43, s | 34.6 | C-2’ | C-5, C-3’/7’ |
| 2’ | - | 137.3 | - | - |
| 3’, 7’ | 7.44, d, *J* = 7.5 Hz | 129.0 | C-4’,6’ | C-1’, C-5’ |
| 4’, 6’ | 7.33, t, *J* = 7.5 Hz | 128.4 | C-3’/7’ | C-2’ |
| 5’ | 7.28, t, *J* = 7.5 Hz | 127.4 | C-4’,6’ | C-3’/7’ |
| N-NH_2_  CH_3_ | 6.03, br s  2.61, s | -  22.9 | -  - | C-3, C-5  - |
|  |  |  |  |  |

Table 4. ^1^H NMR (500 MHz) and ^13^C NMR (125 MHz)

spectral data for compound **14** in (CD_3_)_2_SO

| **Position** | **^1^H** | **^13^C** | **HMBC** | |
| --- | --- | --- | --- | --- |
|  |  |  | ***^2^J*** | ***^3^J*** |
| 2 | - | 164.2 | - | - |
| 4 | - | 158.3 | - | - |
| 5 | 7.47, d, *J* = 6.0 Hz | 121.4 | C-6, C- 9 | C-8 |
| 6 | 7.52, d, *J* = 6.0 Hz | 119.6 | C-5 | C-8, C-9 |
| 8 | - | 167.2 | - | - |
| 9  N-NH_2_ | -  7.76, br s | 113.8  - | -  - | -  - |
| CH_3_ | 2.53, s | 22.6 | - | - |

Table 5. ^1^H NMR (500 MHz) and ^13^C NMR (125 MHz)

spectral data for compound **15** in (CD_3_)_2_SO

| **Position** | **^1^H** | **^13^C** | **HMBC** | |
| --- | --- | --- | --- | --- |
|  |  |  | ***^2^J*** | ***^3^J*** |
| 2 | - | 176.2, 176.2 | - | - |
| 4 | 7.62, dd, *J* = 8.5, 2.0 Hz | 119.0,  119.0 | C-5, C-9 | C-6, C-8 |
| 5 | 7.46, d, *J* = 8.5 Hz | 129.0,  128.9 | C-4, C-6 | C-7, C-9 |
| 6 | 7.14, dd, *J* = 8.5, 2.0 Hz | 111.4,  111.5 | C-5, C-7 | C-4, C-8 |
| 7 | - | 159.3,  159.3 | - | - |
| 8 | - | 123.6, 123.7 | - | - |
| 9 | - | 157.1 | - | - |
| CH_3_ | 2.72, s | 23.9 | - | - |

Table 6. ^1^H NMR (500 MHz), ^13^C NMR (125 MHz) and spectral data for compound **16** in CD_3_OD

| **Position** | **^1^H** | **^13^C** | **HMBC** | |
| --- | --- | --- | --- | --- |
|  |  |  | ***^2^J*** | ***^3^J*** |
| 3 | - | 157.0 | - | - |
| 5 | - | 144.4 | - | - |
| 1’ | 3.83, q, *J* = 7.5 Hz | 38.2 | C-2’ | C-3, C-5 |
| 2’ | 1.32, t, *J* = 7.5 Hz | 14.6 | C-1’ | - |
| CH_3_ | 2.58, s | 23.1 | - | - |

**NMR Spectra of synthesized compounds 13-16**

Figure 4. ^1^H NMR spectrum for compound **13** in (CD_3_)_2_SO (500 MHz)

Figure 5. ^13^C NMR spectrum for compound **13** in (CD_3_)_2_SO (125 MHz)

Figure 6. HMBC NMR spectrum for compound **13** in (CD_3_)_2_SO (500 MHz)

Figure 7. ^1^H NMR spectrum for compound **14** in (CD_3_)_2_SO (500 MHz)

Figure 8. ^13^C NMR spectrum for compound **14** in (CD_3_)_2_SO (125 MHz)

Figure 9. HMBC NMR spectrum for compound **14** in (CD_3_)_2_SO (500 MHz)

Figure 10. ^1^H NMR spectrum for compound **15** in CD_3_OD (500 MHz)

Figure 11. ^13^C NMR spectrum for compound **15** in CD_3_OD (125 MHz)

Figure 12. HMBC NMR spectrum for compound **15** in CD_3_OD (500 MHz)


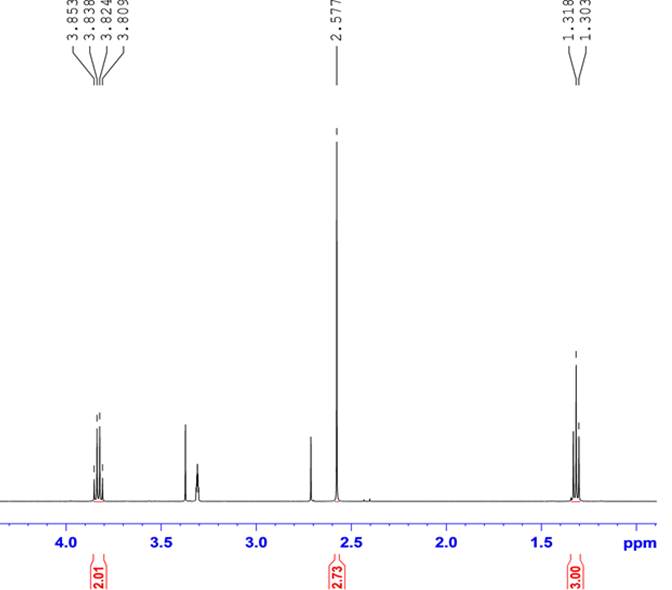


Figure 13. ^1^H NMR spectrum for compound **16** in CD_3_OD (500 MHz)


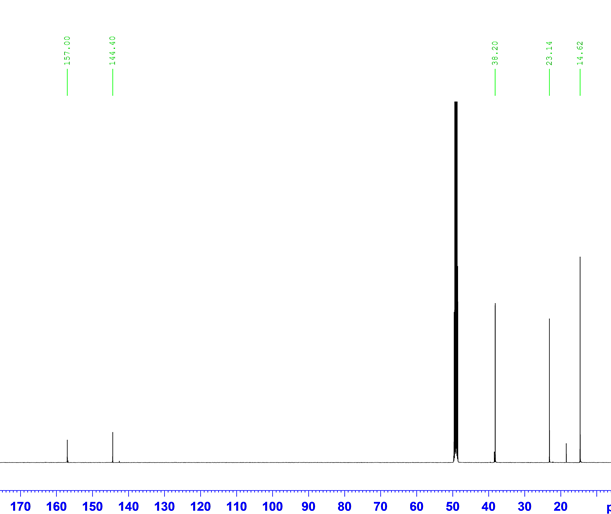


Figure 14. ^13^C NMR spectrum for compound **16** in CD_3_OD (125 MHz)


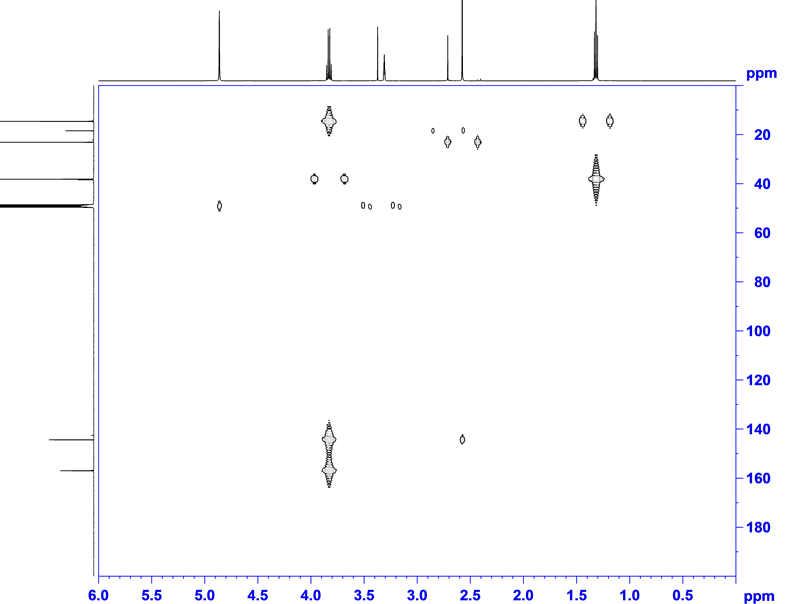


Figure 15. HMBC NMR spectrum for compound **16** in CD_3_OD (500 MHz)
